# Papular dermatitis due to *Leishmania infantum* infection in seventeen dogs: diagnostic features, extent of the infection and treatment outcome

**DOI:** 10.1186/1756-3305-7-120

**Published:** 2014-03-24

**Authors:** Gabriella Lombardo, Maria Grazia Pennisi, Tiziana Lupo, Carmen Chicharro, Laia Solano-Gallego

**Affiliations:** 1 Studio Veterinario Lombardo, via Cavòlo 20, Mascalucia, Catania 95030, Italy; 2 Dipartimento di Scienze Veterinarie, Università di Messina, Polo Universitario Annunziata, Messina 98168, Italy; 3 Istituto Zooprofilattico Sperimentale della Sicilia – Centro di Referenza Nazionale per le Leishmaniosi, via G. Marinuzzi 3, Palermo 90129, Italy; 4 WHO Collaborating Centre for Leishmaniasis, Servicio de Parasitología, Centro Nacional de Microbiología, Instituto de Salud Carlos III Madrid, Spain; 5 Departament de Medicina i Cirurgia Animal, Facultat de Veterinaria, Universitat Autònoma de Barcelona, Cerdanyola 08193 Barcelona, Spain

**Keywords:** *Leishmania infantum*, Dog, Papular dermatitis, Real-time PCR, Leishmanin skin test, Cytology, Culture, Molecular typing, Prognosis

## Abstract

**Background**

This study describes immunological responses, diagnostic features, follow up and treatment outcomes from seventeen dogs with papular dermatitis due to *Leishmania* infection diagnosed by cytology or real time-PCR.

**Methods**

Specific *Leishmania* humoral and cellular immune responses were evaluated by means of an immunofluorescence antibody test in all cases and a delayed-type hypersensitivity (DTH) reaction to leishmanin in eight cases. The extent of infection was studied in several tissues including blood, lymph node, conjunctival and oral swabs, by means of PCR, at the time of diagnosis and during follow-up. Culture was performed on nine dogs from cutaneous lesions and lymph node aspirates and molecular typing was carried out on isolates based on ITS-1, ITS-2 and *Haspb* gene sequencing analysis.

**Results**

Cytological and molecular results from fine needle aspirates of papules were diagnostic in 8 out of 13 (61.5%) cases and in 14 out of 15 dogs (93.3%), respectively. In all dogs, specific anti-*Leishmania* antibody levels were low or absent. Blood and lymph node PCRs and lymph node culture were negative in all dogs. Three out of the nine dogs (33%) were positive by culture from cutaneous lesions. The three isolates were identified as ITS type A, however, polymorphism was observed in the *Haspb* gene (PCR products of 626 bp, 962 bp and 371 bp). DTH response was positive in all tested dogs at the time of diagnosis. The majority of dogs were successfully treated with only N-methylglucamine antimoniate, after which cutaneous lesions disappeared or were reduced to depigmented, flattened scars. All dogs remained seronegative and the majority of dogs were negative by PCR in several tissues during follow-up.

**Conclusions**

This study points out that papular dermatitis due to *L. infantum* is probably an underestimated benign cutaneous problem, associated with a parasite specific cell mediated immunity and a poor humoral immune response. Papular dermatitis is seen in young dogs, and appears to be a mild disease with restricted parasite dissemination and a good prognosis. PCR can be used as a non-invasive method to routinely evaluate papules if *Leishmania* infection is suspected in cases in which parasites are not visualized by cytology.

## Background

*Leishmania* parasites are the causal agents of leishmaniosis, a group of vector-borne protozoan diseases transmitted by phlebotomine sandflies to mammals, including humans. In humans, the disease manifests in different forms, which are usually divided into localized or disseminated cutaneous leishmaniosis (CL), mucocutaneous leishmaniosis and disseminated visceral leishmaniosis (VL) [1].

Canine leishmaniosis (CanL) due to *Leishmania infantum* is a major zoonotic disease endemic in more than 70 countries in the world. It is present in regions of southern Europe, Africa, Asia, South and Central America [1,2] and the USA [3,4]. Dogs are the main reservoir for leishmaniosis caused by *L. infantum*.

The clinical manifestation of *L. infantum* infection in dogs varies widely as a consequence of the numerous pathogenic mechanisms of the disease process, which organs are affected and the diversity of immune responses mounted by individual hosts [5]. The opposite extremes of the broad spectrum of immune responses are characterized by protective immunity that is T cell mediated, or disease susceptibility associated with a marked humoral non-protective immune response and reduced cell mediated immunity [5]. In addition, it is well known that a high proportion of dogs living in endemic areas are subclinically infected with *Leishmania* parasites, while a small proportion of dogs will develop severe disease [5,6]. Therefore, *L. infantum* infection in dogs can manifest as a subclinical infection, as a self-limiting or as severe disease [7].

The main clinical findings based on physical examination in classical CanL includes skin lesions, generalized lymphadenomegaly, progressive weight loss, muscular atrophy, exercise intolerance, decreased appetite, lethargy, splenomegaly, polyuria and polydypsia, ocular lesions, epistaxis, onychogryphosis, lameness, vomiting and diarrhea [8]. Skin lesions are a very common manifestation of the disease [9]. Various cutaneous forms such as alopecia, exfoliative, ulcerative, nodular or sterile pustular dermatitis, nasal or footpad hyperkeratosis, onychogryphosis, paronychia, mucosal or mucocutaneous ulcerations, nodules or masses have been described [9-12]. A distinctive form of papular dermatitis due to *L. infantum* infection was described in dogs living in endemic areas [13-16]. This cutaneous manifestation of *L. infantum* infection is suggestive of a benign form because of the lack of systemic signs and laboratory abnormalities, a good response to therapy with no clinical relapse and on some occasions self-healing of the lesions [13-16]. In addition, papular dermatitis is associated with a *Leishmania*-specific immunocompetence characterized by a predominant parasite specific cellular immunity and low humoral immune response [13-15].

However, information about diagnostic parameters, extent of infection, treatment outcome and prognosis of this distinctive form of papular dermatitis due to *L. infantum* is limited. Therefore, the aim of this study was to further characterize papular dermatitis due to *L. infantum* infection in dogs at time of diagnosis and during follow-up after treatment by: (1) describing clinicopathological findings in a series of clinical cases of papular dermatitis due to *L. infantum* in dogs from Sicily; (2) evaluating the cellular and humoral immunological status in these dogs and the dissemination of infection by sampling several tissue samples such as blood, lymph node, conjunctival and oral swabs and testing using molecular techniques. Culture and molecular typing of isolates were also performed in some cases.

## Methods

### Clinical examination and sampling

Seventeen dogs (16 from Catania and one from Palermo, Italy) were enrolled in this study from September 2008 to September 2012. They were examined for the presence of chronic non-pruritic cutaneous lesions in 7 out of 17 cases (cases 3, 4, 8, 9, 10, 16 and 17) or for routine control in the other cases (cases 1, 2, 5, 6, 7, 11, 12, 13, 14 and 15). A physical examination was performed on each dog. Fine needle aspirates were taken aseptically from one to three cutaneous lesions per case (23 total cutaneous lesions sampled from all cases studied) and from lymph nodes from all dogs. Cutaneous lesions were analyzed by means of cytological examination and real-time polymerase chain reaction (RT-PCR) in 13 and 15 dogs, respectively. Distant or regional lymph nodes were analyzed by means of cytology in 9 dogs and RT-PCR in all dogs. Cytological direct smears were stained with MayGrünwald-Giemsa and evaluated by microscopy. Samples were taken for diagnostic purposes and, therefore, ethical approval was not needed.

Blood EDTA tubes, syringes with fine needle used for aspiration of lymph nodes and cutaneous lesions, tubes with serum and swabs were stored at 4°C and sent to the laboratory (National Reference Centre for Leishmaniosis – Italy) up to 72 hours after sampling for *Leishmania* RT-PCR and in some cases for parasite culture. Real-time polymerase chain reaction for detecting *Leishmania* DNA [17,18] was performed on the following specimens: one or more cutaneous lesions in 15 dogs (20 total lesions tested), peripheral blood and lymph-node aspirates in all dogs, conjunctival swabs in nine cases and oral swabs in eight cases. Sera were tested for IgG antibodies to *L. infantum* antigen by the immunofluorescence antibody test (IFAT) [19].

### IFAT

The IFAT was performed according to Duxbury (1964) [20] and Badaro (1983) [21] with some modifications as previously described [17]. The cut off value was established at 1:80 [19]. Seropositive dogs were ranked in three categories based on range of the IFAT antibody titer as follows: low (1:80–1:320), intermediate (1:640–1:1280) and high positive antibody titers (> 1:1280).

### DNA extraction and *Leishmania* real time PCR on clinical samples

DNA extraction of cutaneous lesions, blood, lymph node, conjunctival and oral swabs and *Leishmania* real time PCR (RT-PCR) were performed as previously described [17].

### Leishmanin skin test

Leishmanin skin test (LST) for the evaluation of a delayed-type hypersensitivity (DTH) reaction was carried out in five cases upon clinical presentation and in three cases during the follow up evaluation (8 to 10 months post treatment). Briefly, 100 μl solution of inactivated suspension of 3×10^8^*L. infantum* promastigotes/mL in 0.4% phenol-saline (kindly provided by Carmen Cañavate Instituto de Salud Carlos III, Madrid, Spain) were intradermally injected in the skin of the groin. Skin reactions were recorded after 48 and 72 h and an induration or erythematous area > 0.5 cm in diameter was considered positive [22,23].

### Culture and molecular typing of isolates

#### Culture

*In-vitro* culture was performed on 9 dogs. Culture was carried out on 12 out of 23 cutaneous lesions sampled and on 9 out of 17 lymph-node fine needle aspirates. Parasites were grown in Tobie agar medium modified by Evans with 15% rabbit blood, 5% fetal bovine serum, 250 μg of gentamicin/mL, and 500 μg of 5-fluorocytosine/mL. The cultures were incubated at 25°C for 7 days. In samples that were negative after 7 days of incubation, 1 mL of the culture sample was subcultured in the medium for a further 10 days.

#### Molecular typing of isolates

DNA was obtained from *Leishmania* cultures using the QIAamp DNA mini kit (QIAGEN).

For molecular typing analysis, the polymorphism of two different target genes was studied: ITS1 and ITS2 (ribosomal DNA internal transcribed spacers) and *haspb* (*k26*) gene (hydrophilic acylated surface protein B).

ITS1 and ITS2 were amplified as described by Kuhls *et al.* (2005) [24]. Direct sequencing of the ITS1 and ITS2 PCR products and the analysis of the data sequences were performed following the protocol described by Chicharro *et al.* (2013) [25].

*Haspb* (*k26*) gene was amplified according to the protocol previously described by Haralambous *et al.* (2008) [26]. PCR products were analyzed by electrophoresis in 2% agarose gel stained with PronaSafe (CONDA) and visualized under ultraviolet light. Product size was estimated with comparison to a 100-bp DNA ladder, and adjusted according to the gene size variability attributed to the number of 42 nucleotide repeated motifs [27,28].

### Treatment and follow up

The treatment protocol that was instituted for patients with papular dermatitis was a non-standard protocol of one dose of N-methylglucamine antimoniate (100 mg/kg SC q24 h) for 25–30 days due to mild disease [7]. Fifteen dogs were treated with N-methylglucamine antimoniate (100 mg/kg SC q24 h) for 25–30 days. Unfortunately, two owners did not agree to perform the treatment protocol mentioned above. In case 11, the treatment offered for the dog was refused by the owner and the dog was rechecked after four months. In case 12, the owner refused antimonial treatment for the dog and the dog was treated with allopurinol (20 mg/kg q24h) for six months.

All dogs were re-visited at the end of therapy (approximately one month after first visit). Unfortunately, monitoring of the dogs after treatment was not so well standardized. Dogs were presented for control visits at different times when owners decided to do a check up at a private veterinary clinic.

Thus, we obtained a long term follow-up (range 6–24 months after clinical presentation) in seven cases based on physical examination and in five of them *Leishmania* infection was assessed by means of IFAT and *Leishmania* real-time PCR on blood, lymph node, conjunctival and oral swabs and in three cases also by means of DTH.

## Results

### Time of diagnosis

Signalment of seventeen dogs studied is listed in Table 1. Half of them were mongrels and their ages ranged from 3.5 to 36 months (mean ± SD 10.3 ± 7.8.months). Eleven out of 17 dogs lived outdoors, 2 indoors and 4 both indoors and outdoors.

**Table 1:** Signalment of dogs, number, distribution of papules and time of duration of lesions

**Case number**	**Breed**	**Age (months)**	**Sex**	**Number**	**Distribution**	**Time of duration of lesions**
1	Mongrel	6	M	Multiple	Nose	Unknown
2	Boxer	6	M	Multiple	Abdomen	Unknown
3	Mongrel	12	F	2	Nose	6 months
4	Labrador retriever	11	F	4	Bridge of the nose	1 month
5	Mongrel	8	M	4	Inner surface of the pinna	Unknown
6	Mongrel	8	M	3	Inner surface of the pinna	Unknown
7	Mongrel	3,5	M	Multiple	Eyelid – Abdomen	Unknown
8	Mongrel	4	F	Multiple	Abdomen	3 weeks
9	Mongrel	4	M	Multiple	Abdomen	3 weeks
10	German Shepherd	6	F	Multiple	Inner surface of pinna - nose	1 month
11	Cirneco dell’Etna	17	M	4	Medial aspect of the tibia	Unknown
12	Cirneco dell’Etna	36	M	1	Inner surface of the pinna	Unknown
13	Cane Corso	18	M	1	Abdomen	Unknown
14	Mongrel	7	F	1	Abdomen	Unknown
15	Mongrel	7	M	1	Abdomen	Unknown
16	Newfoundland	12	M	2-3	Inner surface of the pinna	1 month
17	Rottweiler	10	M	Multiple	Eyelid	1 month

**Figure 1:**
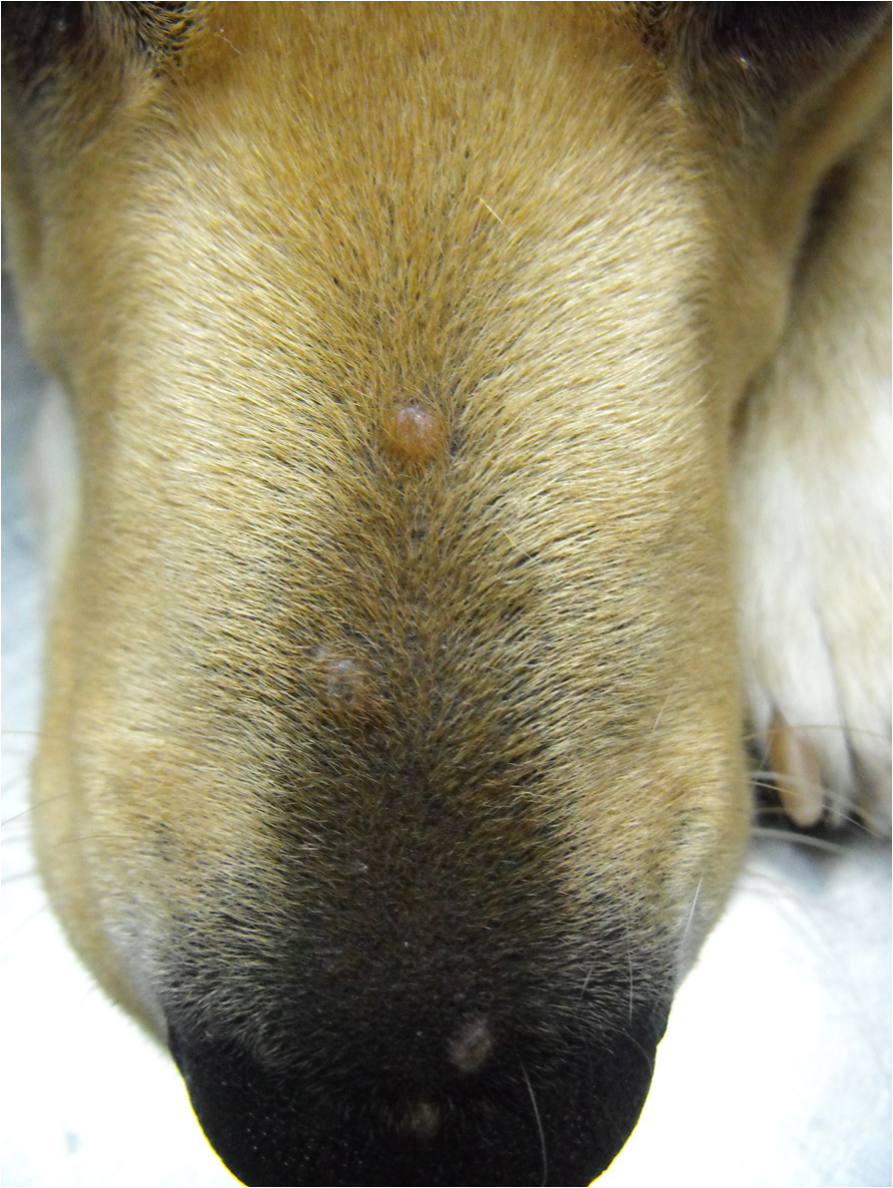
Multiple papular lesions on the nose (case 4).

**Figure 2:**
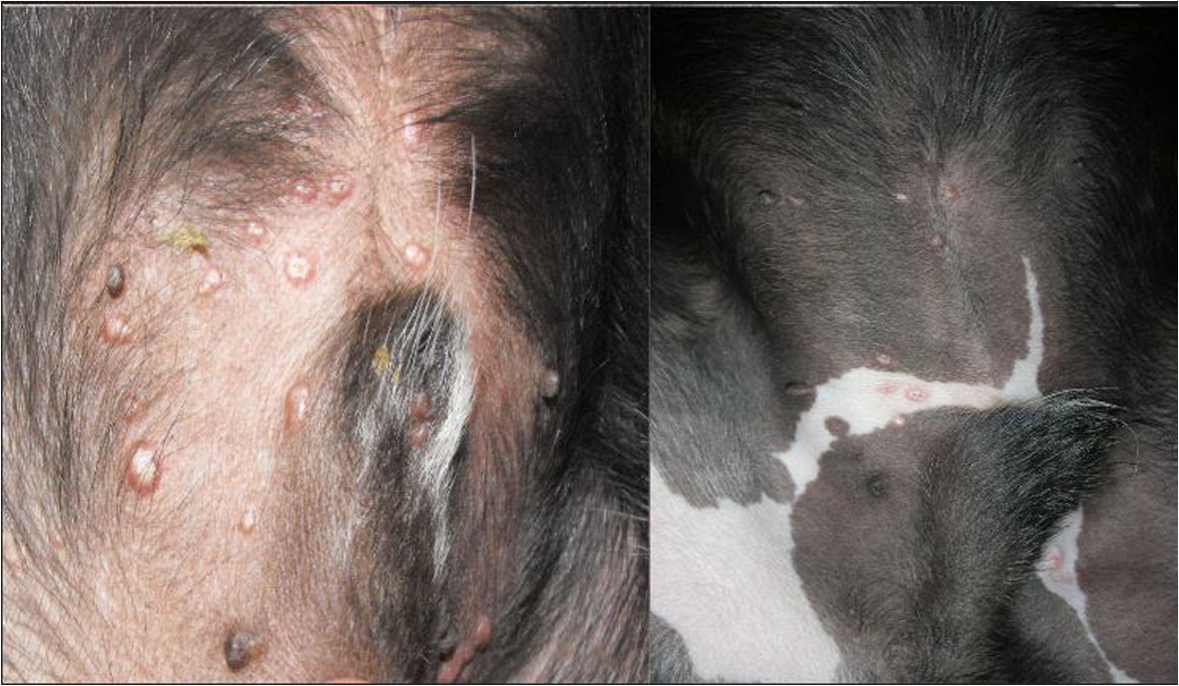
Multiple papular lesions on the skin of abdomen (case 9 on the left and case 2 on the right).

**Figure 3:**
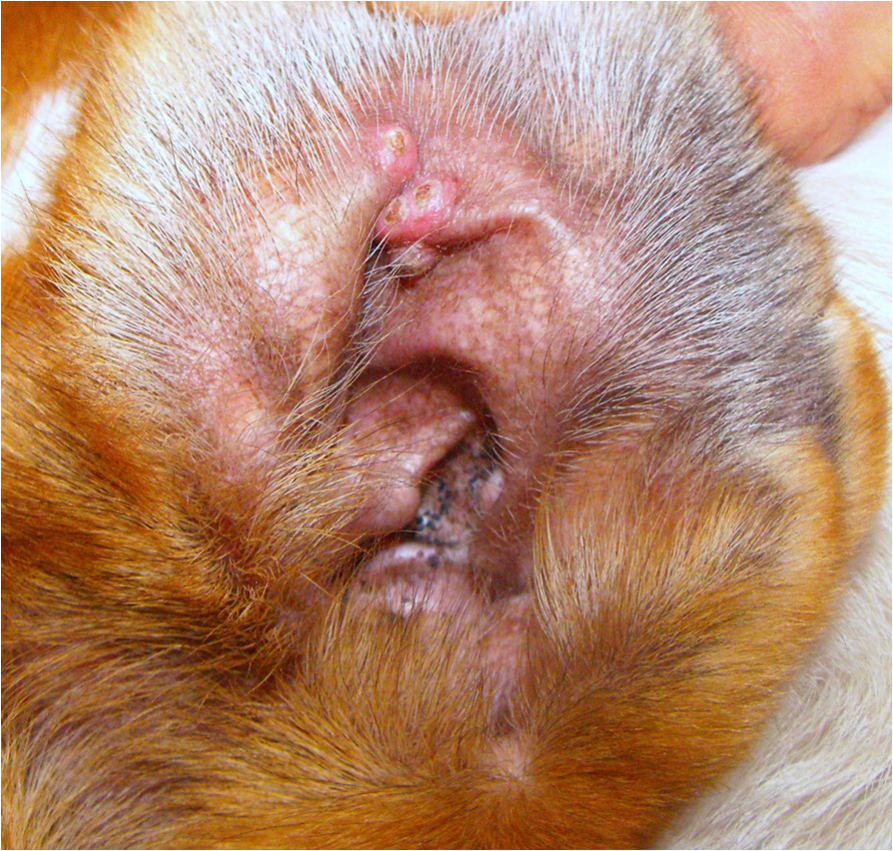
Multiple papular lesions on the inner surface of the pinna (case 5).

On physical examination, solitary or multiple coalescent erythematous, firm and umbilicate non-pruritic papules (diameter 0.2 to 0.5 mm) were observed at various locations on sparsely haired skin areas (Table 1; Figures 1, 2, and 3). In fifteen dogs (88%), papules appeared from September to January and in the two other cases in May and June. In seven cases, cutaneous lesions were chronic from three weeks to six months of duration and, in the other ten cases, lesions were observed as an incidental finding during routine examination. No other abnormalities were found on physical examination, except for two cases where a mild solitary lymphadenomegaly of regional popliteal lymph nodes were noted (cases 8 and 11). PCR was negative in both cases. Cytology of the lymph node revealed reactive hyperplasia (case 8) and no abnormalities (case 11).

**Figure 4:**
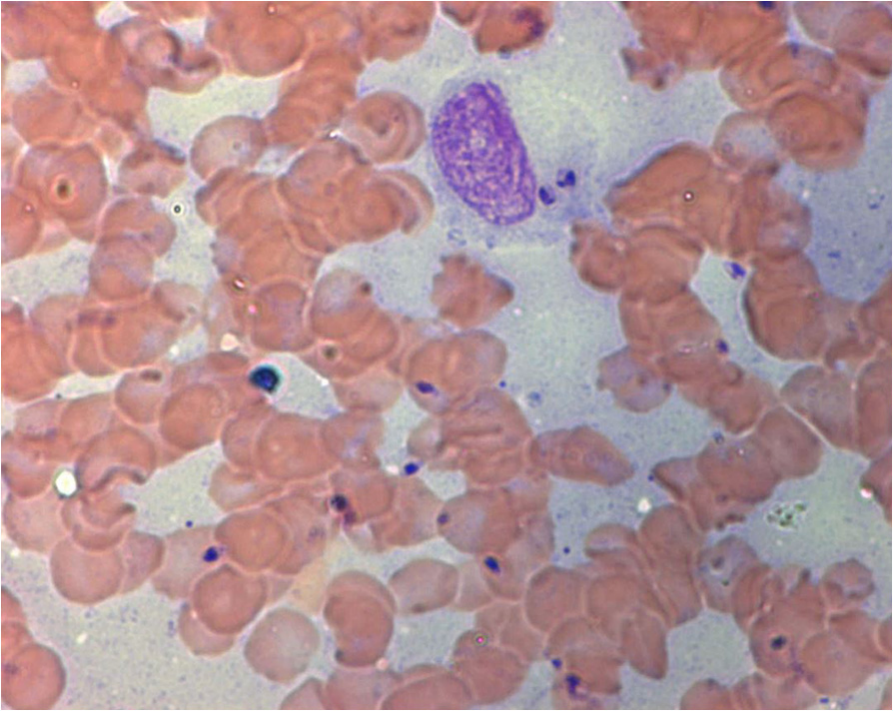
**Numerous extracellular *Leishmania *amastigotes are seen in a hemodiluted sample from a papular lesion from case 10.** Note one macrophage with intracytoplasmatic *Leishmania* amastigotes (Stain May-Grünwald-Giemsa, X1000).

Cytological examination of the papules was diagnostic in 8 out of 13 (61.5%) cases. Variable numbers of intracellular and /or extracellular *Leishmania* amastigotes associated with neutrophilic-macrophagic and/or lymphoplasmocellullar inflammation were observed (Figure 4). In the cutaneous lesions sampled, cytology revealed the presence of *Leishmania* amastigotes in 14 out of 19 lesions (68.4%) (Table 2). RT-PCR performed on cutaneous lesions was positive in 14 out of 15 dogs (93.3%) with positive results in 19 out of 20 lesions (95%) sampled and a parasite load ranging from 20 to 60000 parasite/sample. The diagnosis of *Leishmania* infection was confirmed based on cytology or RT-PCR in all cases. Parasite isolation from cutaneous lesions was obtained only in 3 out of 9 dogs (33.3%) and 5 out of 13 cutaneous lesions sampled (38.4%).

**Table 2:** **Presence of *Leishmania *** **confirmed by cytology, parasite culture, *Leishmania *** **RT-PCR on single or multiple skin lesions from the dogs studied**

**Case number**	**Cytology**	**Culture**	**PCR ( *l */s)**
**1**	+	NP	NP
**2**	+ (a;b;c)	+ (a;c); − (b);	+ (a 60000); + (b 20000); + (c 50000);
**3**	+ (a; b)	- (a; b)	+ (a 8000); + (b 20000);
**4**	+ (a; b; c)	NP	NP
**5**	-	-	+ (200)
**6**	-	-	+ (600)
**7**	-	-	+ (a 5120); + (b 10)
**8**	+	-	-
**9**	-	+	+ (9500)
**10**	+ (a; b)	+(a; b)	+ (a 10000); + (b 6500);
**11**	NP	-	+ (950)
**12**	NP	NP	+ (4000)
**13**	NP	NP	+ (8700)
**14**	+	NP	+ (1350)
**15**	+	NP	+ (2000)
**16**	NP	NP	+ (20)
**17**	-	NP	+ (800)

+/−: presence or absence of *Leishmania* amastigotes by cytology or positive or negative by culture or PCR; NP: not performed; (l/s): *Leishmania*/sample; (a, b or c): results of different lesions sampled.

Results of IFAT, RT-PCR and culture on other tissues are displayed in Table 3. The majority of dogs were seronegative (12 out of 17, 70.6%) and the remaining were low positive with antibody titers ranging from 1:80 to 1:320. Lymph-node cytology revealed reactive hyperplasia in two dogs (case 1 and 8), no abnormalities in five cases (case 3, 4, 6, 10 and 11) and it was non-diagnostic in the remaining two cases (case 2 and 9). Lymph-node culture was negative in all nine dogs.

**Table 3:** **IFAT, *Leishmania *** **RT-PCR and culture results in other tissues at the time of diagnosis and DTH test results at the time of diagnosis and/or during the follow-up**

**Case number**	**IFAT**	**Blood**	**Lymph-node**	**Lymph-node *in-vitro *** **culture**	**Conjunctival swab**	**Oral swab**	**DTH**	**DTH**
		**PCR ( *Leishmania */mL)**	**PCR ( *Leishmania */sample)**			**PCR ( *Leishmania */sample)**	**48 h (cm)**	**72 h (cm)**
					**PCR ( *Leishmania */sample)**			
**1**	-	-	-	NP	NP	NP	2,50^#^	1,75^#^
**2**	-	-	-	-	NP	NP	NP	NP
**3**	-	-	-	-	NP	NP	3,00^#^	3,25^#^
**4**	-	-	-	NP	-	NP	1,60^#^	1,75^#^
**5**	1:80	-	-	-	+ (5)	-	2,25*	1,15*
**6**	-	-	-	-	+ (10)	-	1,25*	2,00*
**7**	-	-	-	-	-	-	NP	NP
**8**	-	-	-	-	+ (70)	+ (3)	1,50*	1,25*
**9**	-	-	-	-	+ (20)	-	1,00*	0,00*
**10**	-	-	-	-	-	-	1,50*	1,75*
**11**	1:160	-	-	-	-	-	NP	NP
**12**	-	-	-	NP	-	-	NP	NP
**13**	1:80	-	-	NP	NP	NP	NP	NP
**14**	1:320	-	-	NP	NP	NP	NP	NP
**15**	1:320	-	-	NP	NP	NP	NP	NP
**16**	-	-	+ (6)	NP	NP	NP	NP	NP
**17**	-	-	-	NP	NP	NP	NP	NP
**Total number of positive results/total number of dogs studied (%)**	5/17 (29.4%)	0/17 (0%)	1/17 (5.8%)	0/9 (0%)	4/9 (44.4%)	1/8 (12.5%)		

+ positive; −: negative; NP: not performed; l/sample: *Leishmania*/sample; *DTH results upon clinical presentation and in some cases # at follow up.

LST was positive in all 5 tested cases at 48 h reading with a mean ± SD diameter of 1.50 ± 0.46 cm (range 1 – 2.25 cm) and was positive in 4/5 of the cases at 72 h reading with a mean ± SD diameter of 1.54 ± 0.40 (range 1.15 – 2 cm).

**Figure 5:**
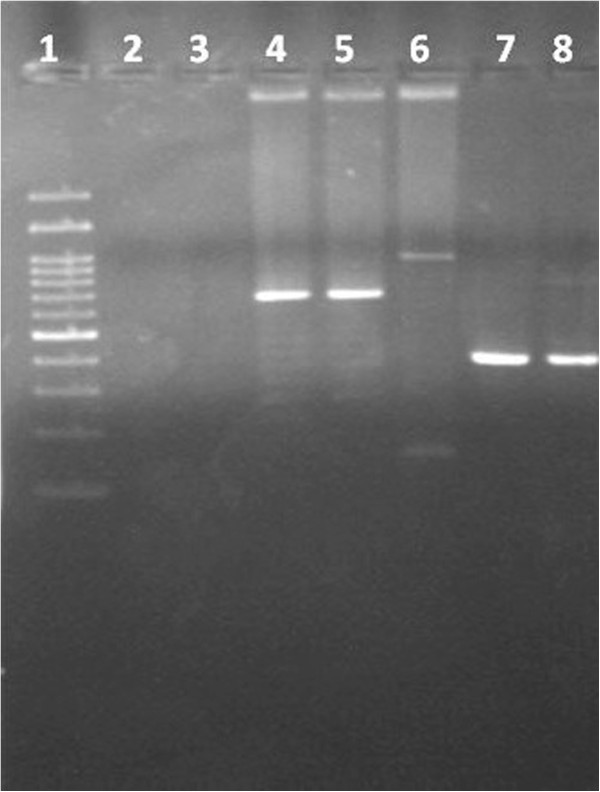
**Haspb –PCR, PCR products were separated by electrophoresis on 2% agarose gels and stained with PronaSafe.** 1.- Mr-100 bp; 2. and 3. - negative control; 4.- case 2 lesion a; 5.- case 2 lesion b; 6.- case 9; 7.- case 10 lesion a; 8.- case 10 lesion b.

Molecular characterization was performed in five *L. infantum* strains isolated from three dogs with skin lesions: case 2 (two samples), case 9 (one sample) and case 10 (two samples) (Figure 5). ITS-1 and ITS-2 were sequenced and all *L. infantum* strains were identified as ITS type A. On the other hand, the polymorphism of the *haspb* (*k26*) sequence was determined by analysis of the size of the PCR products and adjusted according to the *haspb* (*k26*) gene present repeated motifs of 42 nucleotides. Strains isolated from cases 2, 9 and 10 revealed *k26* PCR products of 626 bp, 962 bp and 371 bp, respectively. No differences were observed between strains isolated from the same dog.

### Follow up

The dog that was not treated (case 11) was evaluated four months later. On physical examination, the cutaneous lesion was still present but slightly flattened. In addition, antibody levels decreased from a low positive antibody titer of 1:160 at time of diagnosis to a negative antibody titer of 1:40 at recheck (4 months later). Blood, lymph node and conjunctival swab RT-PCRs were negative 4 months later as well. The dog (case 12), treated only with allopurinol for six months (January- August 2010), remained seronegative and blood, lymph-node and conjunctival swabs were also negative at three and eight months post diagnosis. The dog seroconverted at 10 months (November 2010) (IFAT 1:160) and clinical examination and full CBC and biochemistry profile showed no clinicopathological abnormalities. A treatment with allopurinol was repeated for eight more months (until August 2011). On July 2011, serology was negative. After the second course of therapy, this dog was regularly monitored every six months by IFAT and remained seronegative and clinically healthy in the following year. The last IFAT, performed on March 2012, was still negative.

All dogs treated with meglumine antimoniate were clinically evaluated after 25 days of therapy and the cutaneous lesions had disappeared or changed to a depigmented, flattened scar.

Six out of 17 dogs were only revisited after 25 days of therapy with meglumine antimoniate and then, these dogs were lost to follow up (cases 2, 5, 6, 8, 9 and 13). Five dogs were also assessed for *Leishmania* infection between two and five months post therapy (cases 10, 12, 14 and 16). They remained seronegative (cases 10, 12, 16) or showed a decrease in antibody titers (cases 14 and 15: a diagnostic antibody titer of 1:320 for both cases and post therapy antibody titers of 1:40 and 1:80, respectively). Moreover, blood, lymph node and conjunctival RT-PCRs were also negative. One dog was only clinically evaluated five months post therapy and the dog was in good health with a normal physical examination (case 17).

Long term follow up (6–24 months after therapy) was obtained in seven dogs (cases 1, 3, 4, 7, 12, 14 and 15). Two of them (cases 14 and 15) were assessed only by physical examination and there were no clinical abnormalities. Five dogs were also rechecked for *Leishmania* infection. Three dogs (cases 1, 3, 7) remained negative for both IFAT and RT-PCR performed on blood, lymph-node, conjunctival and oral swabs. One dog (case 4) showed a reactive lymph-node with positive RT-PCR results (n. 300 *Leishmania*/sample) and culture after 9 months but the dog was negative 4 months later by lymph node RT-PCR. A DTH test was performed in three dogs (cases 1, 3, 4) during the follow up period. A positive result was observed in all three cases with a mean ± SD diameter of 2.36 ± 0.71 cm (range 1.6-2.5 cm) at 48 h and a mean ± SD diameter of 2.25 ± 0.86 (range 1.75-3.25 cm) at 72 h.

## Discussion

This study describes seventeen cases of papular dermatitis due to *Leishmania* infection in dogs living in a highly endemic area for canine leishmaniosis (Sicily) and is the largest published case series concerning this cutaneous clinical manifestation of *Leishmania* infection. Nodular lesions caused by *Leishmania* infection were previously described to include lesions of variable size from a few millimetres to 10 cm in diameter [10]. More recently, papular and nodular dermatitis were differentiated into two distinct dermatological forms of cutaneous lesions caused by *L. infantum* as described in the literature [13-15]. In fact, the lack of progression of these persistent papules to nodules characterizes clinically this benign form of dermatitis due to *Leishmania* infection in the present study and in previous studies [13-16]. It is noteworthy that in 58% of this case series the papular lesions were not noticed by the owner and were incidentally found during the physical examination and, therefore, these cutaneous lesions might be underdiagnosed. The young age, the distribution and evolution of papular lesions, and the clinical findings of these dogs are similar to those reported in the previous studies [13-16].

Persistent, solitary or multiple coalescent erythematous and firm umbilicate non-pruritic papular lesions were located on sparsely haired skin areas and the majority of dogs (82%) were one year of age or less. The presence of papular lesions in young dogs might be the direct consequence of the first contact of an immunocompetent host with the parasite inoculated by sandflies in the skin. This occurred mainly from September to January potentially some months after the end of the classical sandfly season previously reported by Noli *et al*., (2006) [14]. We hypothesize that the onset of cutaneous lesions at the end of or after the sandfly season might occur after a silent phase of parasite amplification, as described in mice experimentally infected by intradermal inoculation with a low dose of *Leishmania major* promastigotes [29]. However, a prolonged duration of *P. pernicious* activity until November has been demonstrated in some Italian provinces [30]. The two most prevalent sandfly species found in Sicily are *Phlebotomus perfiliewi* and *P. pernicious,* however, studies about seasonal activity of sandflies in Sicily are lacking [30-32]. Other studies reported papular dermatitis in summer months [13-15] as we observed in two cases in our study.

In this study, papular dermatitis was associated with no other clinical signs (excluded two cases of mild regional lymph node enlargement), absence of or low levels of humoral immunity and a predominant parasite specific cellular immunity in all dogs studied as previously reported [15]. In addition, all dogs treated with only meglumine antimoniate that were followed-up in the long term promptly cured with antimonial therapy, did not develop any other clinical manifestations, did not seroconvert and they probably contained the parasite infection, suggesting that papular dermatitis is associated with competent specific immunity and has an excellent prognosis. The only dog treated with a six month course of therapy with allopurinol seroconverted with a low antibody level which required a second course of therapy but remained clinically healthy and reverted to a negative persistent antibody status after the second course of therapy. Thus, papular dermatitis is considered a mild clinical manifestation of this infection (stage I) when accompanied with negative or low antibody levels and no laboratory abnormalities [7]. Similar benign forms of cutaneous leishmaniosis have been described in human beings and horses [33-35]. In human beings, this is the most common form of disease caused by *L. infantum* in Europe. Patients present one or more localized and ulcerated skin lesions, which may also self-cure and are mainly associated with a positive delayed type hypersensitivity (DTH) response [1] as reported previously in dogs with papular dermatitis [13] and as described in the present study in one dog which self-cured. *Leishmania infantum* has also been reported as a causative agent of cutaneous leishmaniosis in the horse in various European countries, including Spain, Portugal and Germany [33-35]. The skin lesions reported were solitary or multiple papules or nodules, most commonly present in the head, pinnae, scrotum, legs and neck. Even in horses self-healing from the cutaneous lesions after a short period of time there is an association with a strong *Leishmania*-specific cellular immunity and a low humoral response [33,34].

As mentioned previously, papular dermatitis due to *Leishmania* in young dogs resembles the typical form of localized cutaneous leishmaniosis, mainly seen in children between 10 and 15 years of age. The lesion begins as a single, asymptomatic, pink or red papule, which is 3–5 mm in diameter, found at the site of the sandfly bite. However, unlike the canine species, the papule slowly evolves to a firm, inflamed, smooth nodule that enlarges progressively and eventually ulcerates, four to twelve weeks after its appearance. Five to twelve months after the initial appearance, the nodulo-ulcerative lesions begin to regress from the center and resolve completely, leaving a scar [36]. In addition, it seems that in human localized cutaneous leishmaniosis the number of parasites present in the lesions is inversely proportional to the duration of the lesion [37]. Unfortunately, we could not corroborate this finding in the present study due to the limited number of dogs where duration of the lesions was known.

This study evaluated, for the first time, the extent of dissemination of *Leishmania* infection in dogs with papular dermatitis as only clinical manifestation. In most of the dogs studied, the only evidence of parasitic infection was in the cutaneous lesions and not in other tissues sampled such as blood, lymph node, oral or conjunctival swabs, at both time of diagnosis and follow-up. Therefore, dogs with papular dermatitis and with the immunological profile described in the present study appear to contain the *Leishmania* infection in the skin.

Some dogs were positive by conjunctival swab PCRs even if they had a very low parasite load. PCR on conjunctival swabs has shown good sensitivity (92%) and specificity (100%) in the diagnosis of classical severe CanL [38,39]. Experimentally infected dogs were found to be positive by PCR on conjunctival swabs as early as 45 days post infection (83%) and before seroconversion [38-40]. In addition, a previous study suggested the use of PCR on conjunctival swabs as a non-invasive alternative diagnostic method to lymph node PCR [17]. These findings suggest that *Leishmania* infection in some dogs is probably not limited to the site of inoculation even in dogs that might restrict the infection and, therefore, there are other tissues where parasite infection might be present.

Only one dog was positive on oral swab PCR. The detection of *Leishmania* DNA in oral swabs has been described in humans with visceral leishmaniosis and in asymptomatic individuals [41] as well as in dogs without any evident oral lesions [17]. A recent study performed in Europe reported low sensitivity of *Leishmania* PCR in oral swabs in both infected or sick dogs without oral lesions (at least stage II of disease) [17,42], while in Brazil a good sensitivity was found [43]. Differences between these studies might be due to the existence of other *Leishmania* species in Brazil that can be detected by PCR and the inclusion of sick dogs with oral or mucosal lesions in the study carried out in Brazil [43]. Evidence to date indicates that in Europe, the sensitivity of the detection of *Leishmania* DNA in oral swabs for diagnosis of subclinically infected or sick dogs appears to be low and not very useful [17,42].

In this study, RT-PCR has been tested as a diagnostic tool to detect *Leishmania* infection in fine needle aspiration of cutaneous lesions and was found more sensitive than cytological evaluation. In the past, the presence of *Leishmania* parasites in skin lesions was investigated using cytological examination, *in vitro* culture or immunohistochemistry on skin biopsy samples. The low number of amastigotes in some samples can make cytological and histopathological diagnosis difficult [14,44,45]. The application of immunohistochemical techniques has given better results in the diagnosis of cutaneous leishmaniosis despite the limitations of this technique due to the presence of false negative cases [45]. In some of our cases, cytological evaluation was not diagnostic probably due to either the small size of the lesions or the hemodilution of the sample. Recently, PCR on paraffin-embedded, frozen skin biopsy specimens or dermal scraping for detection of *Leishmania* amastigotes in cutaneous lesions has been described in both veterinary and medical literature. PCR sensitivity ranged from 81 to 100%, when compared to the presence of *Leishmania* parasites in culture, cytological and histopathological examination [46-49]. In a recent study, a PCR performed on serous material collected by puncture from cutaneous lesions in human patients with CL demonstrated good concordance with parasitological results [50]. It is worthy to mention that although a positive PCR result from a papule from a dog living in an endemic area does not necessarily mean that *Leishmania* is the cause of the papule, due to the high frequency of positive PCR in the skin of infected dogs [6]. However, the list of differential diagnoses of papules in dogs is low and, therefore, when the clinical picture and cytological or histological findings are consistent with *Leishmania* infection, the most likely cause of papules is *Leishmania* infection, even if diagnosed by PCR.

There is limited information on the use of *in vitro* culture for the diagnosis of *Leishmania* infection in dogs in a clinical setting. The majority of studies have evaluated the use of culture from lymph node, bone marrow and spleen samples [51-54], while investigations on the use of culture from skin or blood samples are less common [55,56]. Although culture is a highly specific diagnostic test, this technique is less frequently used at present for various reasons including delay in results, susceptibility to microbiological contamination and in general poor sensitivity compared to molecular techniques. In addition, molecular and/or isoenzymatic techniques are required for species identification [51]. In the present study, only 33% of cultured cutaneous lesions were positive, confirming the low sensitivity of this technique. It is important to highlight that a positive skin culture is most likely in dogs with clinical illness [55], even with mild disease, as shown from results of the dogs evaluated in the present study. A positive skin culture is less likely in subclinically infected dogs [57].

Although *in vitro* culture appears not to be useful for diagnostic purposes, molecular typing of *L. infantum* isolates is an extremely useful taxonomic tool that contributes to better understanding of the epidemiological and pathogenetic aspects of leishmanioses. Molecular genotyping of *L. infantum* strains isolated from papular dermatitis showed that all five samples analysed were *L. infantum* ITS type A, which is a very common ITS sequence type mainly described in human isolates with VL and CL from the Mediterranean basin, including Italy [24,25]. To the best knowledge of the authors, only two previous canine isolates (MON-108 from France and MON-77 from Spain) have undergone molecular typing by ITS sequence analysis resulting in ITS type A and type B/A, respectively [24]. *Haspb* characterization revealed polymorphism in the present study with three different gene sizes (626, 962 and 371 bp) for three different dogs. The 626 bp *haspb* gene size is commonly described in the Mediterranean basin both in humans and dogs [25,26]. The 962 bp *haspb* gene size is also reported in the Mediterranean basin in humans mainly with VL [25]. The 385 bp *haspb* gene size is encountered in non MON-1 canine isolates from the Mediterranean basin including Sicily [26] and it is probably identical to the same fragment as the 371 bp *haspb* gene size described in the present study due to the fact that, in the previous study, adjustment according to the gene size variability attributed to the number of 42 nucleotide repeated motifs was not performed after electrophoresis [26]. Multilocus enzyme electrophoresis, the classical reference method for *Leishmania* typing [58] was not performed due to the fact that it is laborious and expensive and, therefore, in the present study, it was not possible to match the MON system with the molecular typing results. Unfortunately, the number of analysed strains was very low and it was not possible to establish any type of correlation between the genotypes identified and this distinctive form of papular dermatitis in dogs.

## Conclusions

In conclusion, papular dermatitis due to *L. infantum* is an underestimated clinical occurrence associated with a predominant parasite specific cellular immunity and low humoral response, seen in young dogs in endemic areas. This clinical entity is confirmed to be a mild cutaneous clinical manifestation of *Leishmania* infection with restricted parasite dissemination and excellent prognosis when treated for one month with antimonials. Cytological evaluation of papules in dogs living in endemic areas is a useful technique to evaluate inflammation and presence or absence of *Leishmania* amastigotes. However, due to the higher sensitivity of RT-PCR technique in cutaneous aspirates, RT-PCR can be used as a non- invasive method to routinely evaluate persistent papules, if *Leishmania* infection is suspected, in cases in which parasites are not visualized by cytology.

## Abbreviations

CanL: Canine leishmaniosis; CL: Cutaneous leishmaniosis; DTH: Delayed-type hypersensitivity; haspb gene: Hydrophilic acylated surface protein B; IFAT: Immunofluorescence antibody test; ITS: Ribosomal DNA internal transcribed spacers; LST: Leishmanin skin test; RT-PCR: Real-time polymerase chain reaction; VL: Disseminated visceral leishmaniosis.

## Competing interests

The authors declare that they have no competing interests.

## Authors’ contributions

GL designed the study, collected the cases, contributed with data analysis and interpretation and wrote the manuscript. TL performed serological, molecular analysis and culture. CC performed molecular typing of isolates and contributed with data analysis and interpretation. MGP contributed with data analysis and interpretation and performed a critical revision of the manuscript. LSG designed the study, contributed with data analysis and interpretation and wrote the manuscript. All authors read and approved the final version of the manuscript.
